# Alu- and 7SL RNA Analogues Suppress MCF-7 Cell Viability through Modulating the Transcription of Endoplasmic Reticulum Stress Response Genes

**Published:** 2013

**Authors:** D.N. Baryakin, D.V. Semenov, A.V. Savelyeva, O.A. Koval, I.V. Rabinov, E.V. Kuligina, V.A. Richter

**Affiliations:** Institute of Chemical Biology and Fundamental Medicine, Siberian Branch of the Russian Academy of Sciences, Lavrentiev Ave., 8, Novosibirsk, Russia, 630090; Novosibirsk State University, Pirogova Str., 2, Novosibirsk, Russia, 630090

**Keywords:** Alu-repeats, Alu-RNA, 7SL RNA, MCF-7 human breast adenocarcinoma cells

## Abstract

11% of the human genome is composed of Alu-retrotransposons, whose transcription by RNA polymerase III (Pol III) leads to the accumulation of several hundreds to thousands of Alu-RNA copies in the cytoplasm. Expression of Alu-RNA Pol III is significantly increased at various levels of stress, and the increase in the Alu-RNA level is accompanied by a suppression of proliferation, a decrease in viability, and induction of apoptotic processes in human cells. However, the question about the biological functions of Pol III Alu-transcripts, as well as their mechanism of action, remains open. In this work, analogues of Alu-RNA and its evolutionary ancestor, 7SL RNA, were synthesized. Transfection of human breast adenocarcinoma MCF-7 cells with the Alu-RNA and 7SL RNA analogues is accompanied by a decrease in viability and by induction of proapoptotic changes in these cells. The analysis of the combined action of these analogues and actinomycin D or tamoxifen revealed that the decreased viability of MCF-7 cells transfected with Alu-RNA and 7SL RNA was due to the modulation of transcription. A whole transcriptome analysis of gene expression revealed that increased gene expression of the transcription regulator* NUPR1 *(p8), as well as the transcription factor *DDIT3 *(CHOP), occurs under the action of both the Alu- and 7SL RNA analogues on MCF-7 cells. It has been concluded that induction of proapoptotic changes in human cells under the influence of the Alu-RNA and 7SL RNA analogues is related to the transcriptional activation of the genes of cellular stress factors, including the endoplasmic reticulum stress response factors.

## INTRODUCTION

45% of the human genome is composed of mobile elements, of which Alu-repeats are the most numerous, ~ 1.1 X 10^6^ copies, which accounts for 10.6 % of nuclear DNA [1, 2]. In a variety of Alu-repeats of primates, several subfamilies are identified and classified into three main groups: AluJ, AluS, and AluY [3]. The copy number of representatives of evolutionarily ancient AluJ-repeats, which emerged in the genome about 80 million years ago, and intermediate AluS subfamilies (about 40 million years ago), has not increased in the human genome. AluY subfamily repeats (> 20 million years ago) still remain transpositionally active [4].

It is known that the formation of new copies of Aluand related SINE -repeats in the genome of mammalians occurs by a retrotransposition mechanism, which comprises a step for the production of RN A copies of SINE -DNA. Evolutionarily significant variations in the genome occur due to the “successful” events of retrotransposition of repeats in germ cells [5, 6].

**Fig. 1:**
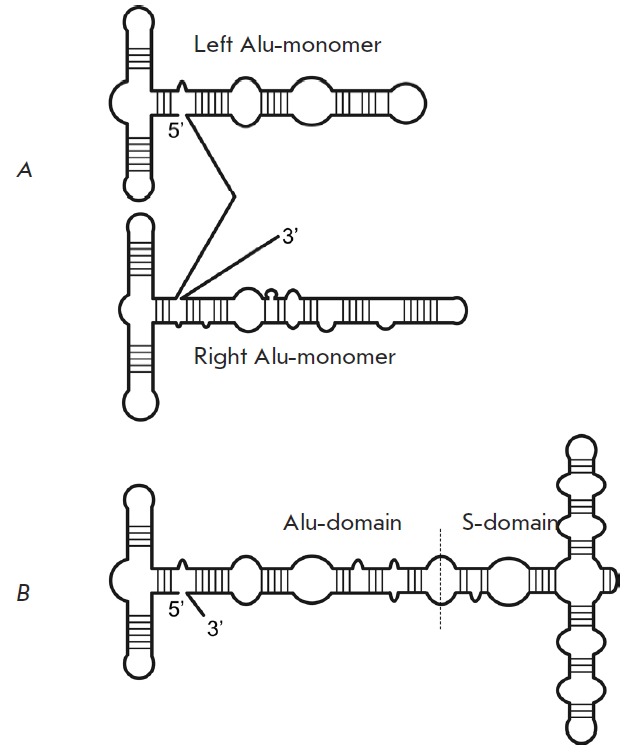
A schematic representation of the secondary structure of Alu-RNA (A) and 7SL RNA (B) according to [12]

However, it is known that RN A copies of genomic Alu-repeats (Alu-RN A) are present both in germ and in somatic human cells [7]. Alu-RN As are synthesized by RN A polymerase III (Pol III) [8] and are a set of RN A copies of the “ancient,” transpositionally inactive Alurepeats of the subfamilies J and S, and transpositionally active AluY [7, 9, 10]. Alu-RN As, as well as their evolutionary ancestor, 7SL RN A, are synthesized in the nucleus and are then transported into the cytoplasm. Some Alu-transcripts undergo 3’-endonuclease processing to yield the truncated forms, scAlu-RN As, represented by the “left” Alu monomers (Fig. 1 A). Along with the truncated Alu-transcripts, unprocessed forms are determined in cells. The latter are represented by Alu- RN A, which includes the “left” and “right” monomers, and the 3’-terminal poly-A-sequence (Fig. 1 A) [10, 11]. The number of full-length Pol III Alu-transcripts is ~ 102–103 molecules per cell. Regulation of Alu-RN A expression in human cells differs from that of other Pol III-transcripts. Thus, a translation inhibitor, cycloheximide, and heat shock increase the expression of Alu- RN A to a greater extent compared with the expression of other Pol III-transcripts, such as 7SL, 7SK, 5S and U6 RN As [8]. The permanent presence of full-length Alu transcripts in the cytoplasm, as well as an increase in the expression of these RN As under stress, on one hand, indicates that Alu-RN A is a closely controlled endogenous factor of mutagenesis and, on the other hand, makes it possible to suggest that Alu-RN As are regulators of vital cellular processes [12].

Earlier K. Sakamoto *et al*. [13] demonstrated that transfection of HeLa cells with DNA constructs containing transcriptionally active Alu-repeats, as well as with constructs encoding 7SL RN A, causes suppression of DNA replication, inhibits translation, and provides an antiproliferative effect. It was found that transfection of human embryonic kidney HEK 293 cells with DNA encoding Alu-repeats leads to specific activation of the expression of the reporter genes, presumably due to direct inhibition of the dsRN A-activated protein kinase PKR by Alu-RN A [14]. It was shown later [15] that activation of reporter gene expression in the presence of Alu-RN A was induced by a decrease in the lag period of translation of newly synthesized mRN As and was not associated with inhibition of PKR. However, a new molecular mechanism, by which Alu-RN A might affect the translation initiation of newly synthesized mRN As, was not proposed.

J. Hasler and K. Strub assumed that the participation of Pol III Alu-transcripts in cellular processes was related to their structural similarity with 7SL RN A (*Fig. 1*) [12, 16]. Like 7SL RN A, Alu-RN A interacts with the proteins of the signal recognition particle (SRP) [17, 18]. The ability of Alu-RN A to modulate translation is attributed to its interaction with SRP9/14 proteins: it has been shown that Alu-RN A activates translation, but Alu-RN A in complex with SRP9/14 inhibits the *in vitro *translation of total mRN A in HeLa cells in wheat germ extracts [16].

It has been demonstrated *in vitro *that Alu-RN A directly interacts with the catalytic subunit of human RN A polymerase II (Pol II) and inhibits the activity of the complex Pol II-TBP-TFIIB-TFIIF at a step of transcription initiation [19, 20]. These data suggest that Alu- RN A is a nonspecific regulator of mRN A transcription in human cells [19].

Recently, in a study on the molecular and cellular mechanisms of the geographic atrophy of the retina (one of the main reasons for a decrease in visual acuity and blindness in people older than 50 years) it was found that retinal pigment epithelial cell death is accompanied by a decrease in the *DICER1 *gene expression and by the accumulation of the Pol III AluSc-transcript in these cells [21]. It was shown that the key enzyme in posttranscriptional microRN A processing, Dicer1 RN ase, hydrolyzes Alu-RN A *in vitro*. A decreased expression of *DICER1 *leads to the accumulation of AluSc-RN A, which in turn suppresses the viability and induces the apoptotic death of the epithelial cells of the retina [21]. A molecular mechanism for the cytotoxic action of Alu- RN A in pigmented epithelial cells has been suggested. It includes the generation of reactive oxygen species by mitochondria, activation of NLRP3-inflammasomes, as well as activation of the MyD88-signaling cascade [22]. Thus, it is the increase in the Alu-RN A expression level that is considered as the main cause of cell death in the geographic atrophy of the retina. However, the question as to why Alu-RN A causes reactive oxygen species formation remains open [21, 22].

In this work, the AluYa5-RN A and 7SL RN A analogues were synthesized and a comparative analysis of their effect on the viability and activation of proapoptotic processes in MCF-7 human breast adenocarcinoma cells was performed. The effect of the Alu-RN A and 7SL RN A analogues, along with cytostatics (inhibitors of replication, transcription, translation, and cellular transport), on MCF-7 cells was also analyzed. It has been established that the proapoptotic processes induced in MCF-7 cells by the Alu-RN A and 7SL RN A analogues are modulated by tamoxifen and actinomycin D. The results of the whole transcriptome analysis of gene expression variation in cells transfected with the Alu-RN A and 7SL RN A analogues allow us to put forward a new mechanism of the cytotoxic action of these RN As based on the activation of the ER stress response genes *NUPR1*, *DDIT3*, *FOXRED2*, and *ASNS*.

## EXPERIMENTAL

**Reagents**

The following reagents were used in this work: MTT – 3-(4,5-dimethylthiazol-2-yl)-2,5-diphenyl -2H-tetrazolium bromide (Sigma, USA); Trizol, Lipofectamine 2000 (Invitrogen, USA); Taq-polymerase, T7-RN Apolymerase (Fermentas, USA); propidium iodide, JC-1 indicator, staurosporine (Sigma, USA); annexin V–FITC conjugate (BD Pharmingen, USA); cisplatin (LEN S-Farm, Russia); cycloheximide, actinomycin D (AppliChem, Germany); interferon α (Microgen, Russia); methotrexate, monensin (Sigma, USA); tamoxifen (Veropharm, Russia), the human recombinant tumor necrosis factor α (State Research Center of Virology and Biotechnology VECT OR, Novosibirsk, Russia), reverse transcriptase MoMLV, ribonucleoside triphosphates, deoxyribonucleoside triphosphates, and T4-polynucleotide kinase (Biosan, Novosibirsk, Russia). Deoxyribooligonucleotides were synthesized in the Laboratory of Medicinal Chemistry, Institute of Chemical Biology and Fundamental Medicine (SB RAS).

**Synthesis of the Alu- and 7SL RNA analogues**

To obtain DNA templates, which are PCR products encoding the Alu- and 7SL RN A analogues under the T7 phage RN A polymerase promoter, genomic DNA of MCF-7 cells was amplified with the following primer pairs (T7-RN A polymerase promoter is shown with lowercase letters): AluYa5, chr6:104,183,151-104,183,559: 5’-ATTT GATTC GGTT ATTTCC AAGA-3’, 5’-atgcagctaatacgactcactataggGAGAGTCTC AGCT ACAGAATT GAA-3’; 7SL, chr14:50,329,268- 50,329,585: 5’-AAGAGACGGGGTCTC GCT AT-3’, 5’-atgcagctaatacgactcactataggg- TTC GCAGCGTCTCC GACC -3’.

DNA templates were purified by electrophoresis in a 10% polyacrylamide gel (PAGE) under native conditions. DNA was eluted from the gel in the presence of 100 mM NaAc and then re-precipitated with 70% ethanol.

The human AluYa5-RN A and 7SL RN A analogues were synthesized in a buffer containing 40 mM Tris- HCl (pH 8.0), 6 mM MgCl_2_, 10 mM DTT , 10 mM NaCl, 2 mM spermidine, 2 mM NT P, and 30 units T7 phage RN A polymerase at 37 °C for 2 hrs. DNA templates were digested in the presence of 1 unit DNAse I at 37 °C for 40 min, and then DNAse I was inactivated by incubation at 65 °C for 15 min.

Purification of the AluYa5-RN A and 7SL RN A analogues was performed on a MiLiChrom A-02 chromatography system (EcoNova, Russia) with re-precipitation with 70% ethanol in the presence of 100 mM NaAc. The primary structure of the analogues was confirmed by RN A reverse transcription, cDNA amplification, and sequencing by the Sanger method on an automatic sequenator, ABI 3730XL Genetic Analyzer (SB RAS Genomics Core Facility).

**Analysis of the viability of MCF-7 cells transfected with the Alu- and 7SL RNA analogues**

Human breast adenocarcinoma cells were cultured in a IMDM medium supplemented with 10 mM Lglutamine, 100 u/ml penicillin, 0.1 mg/ml streptomycin, 0.25 μg/ml amphotericin, and a 10% fetal bovine serum at 37 °C in a 5% CO_2_ atmosphere. The cell number was counted in the Goryaev chamber.

MCF-7 cells were cultured in a 96-well plate until a 60-70% confluent monolayer was formed. The cells were transfected with 1 μg/ml RN A in a complex with Lipofectamine (Invitrogen, USA) according to the manufacturer’s protocol and were incubated for 24 or 72 hrs as indicated in the legends to Tables. The medium was added with MTT to a final concentration of 0.7 mg/ml and was incubated at 37 °C for 45 min. The medium was removed, MTT formazan was dissolved in isopropyl alcohol, and the solution’s optical density was determined by absorbance at λ=570 nm with the control at λ=620 nm using an Apollo LB 912 8 multichannel spectrophotometer (Berthold Technologies).

**Analysis of proapoptotic changes in MCF- 7 cells by flow cytofluorometry**

MCF-7 cells transfected with the Alu- and 7SL RN A analogues and the control cells incubated in the medium with Lipofectamine without RN A were washed three times with PBS and were incubated in the presence of 0.1 mg/ml trypsin at 37 0C for 5 min. To analyze cell membrane changes, a cell suspension was incubated in the presence of 4.5 μg/ml propidium iodide and annexin V – FITC conjugate according to the manufacturer’s protocol (BD Pharmingen, USA). A cell suspension was incubated with 2.5 μg/ml JC-1 to analyze the changes in the mitochondrial transmembrane potential (δΨ). Preparations were analyzed by flow cytofluorometry on a Beckman Coulter FC 500 device according to the method described in [23]. MCF-7 cell preparations incubated with a 5 μg/ml tumor necrosis factor α or with 1 μM staurosporine for 24 hrs were used as a positive control of the proapoptotic changes.

**Analysis of variations in the transcriptome of MCF-7 cells using Illumina chips**

MCF-7 cells were transfected with 1 μg/ml Alu-RN A or with 1 μg/ml 7SL RN A and incubated at 37 °C in a 5% CO_2_ atmosphere for 24 hrs. Cells incubated with Lipofectamine without RN A under the same conditions were used as a control. Hybridization of the total RN A of MCF-7 cells on HT-12 Illumina chips was performed on the basis of Genoanalytika, CJSC (Moscow). The differential analysis of the variations in gene expression was performed using the Illumina custom algorithm with data normalization by the rank invariant method. The parameter Detection_ Pval < 0.05 was used to interpret the results of the differential analysis of gene expression variations. Upon interpretation of the data on an increase in the gene expression under the influence of Alu-RN A, transcripts for which the structure of hybridization probes (Illumina PROBE_SEQUENCE ) contained direct sequences of Alurepeats were excluded from consideration.

Sampling verification of the whole transcriptome analysis results was performed with the real-time RT - PCR method using the following primer pairs:* PSPH *- 5’-ATGATTGGAGATGGTGCCAC-3’, 5’-CAGTGATATACCATTTGGCG-3’;* DDIT3 *- 5’-GACCTGCAAGAGGTCCTGTC-3’, 5’-AAGCAGGGTCAAGAGTGGTG-3’;* MTHFD *- 5’-TGTAGGACGAATGTGTTTGG-3’, 5’-AACATTGCAATGGGCATTCC-3’;* TDP1 *- 5’-CTCATCAGTTACTTGATGGC-3’, 5’-TGACTTCCTTGAAAGCGTCC-3’;* ZNF682 *- 5’-AAGCCAGAACTGATTAGCCG-3’, 5’-AAGGTCTTCAGTGTAATGAG-3’;* CEBPG *- 5’-CGCTCGGAGTGGAGGCCGCC-3’, 5’-CAGGGTGATCAATGGTTTCC-3’.* GAPDH *and *HPRT *mRN As were used as normalization control [24].

## RESULTS AND DISCUSSION

**Influence of the Alu- and 7SL RNA analogues on the viability of human breast adenocarcinoma MCF-7 cells**

The action of Alu-RN A and its evolutionary ancestor, 7SL RN A, on human cells was analyzed using an analogue of Alu-RN A, a transcript of human genomic repeat AluYa5, as well as using an analogue of 7SL RN A.

It was found that transfection of MCF-7 human breast adenocarcinoma cells with the Alu- and 7SL RN A analogues caused substantial morphological changes: condensation of the cytoplasm and nucleus, degradation of membrane contacts, and cell detachment from the plastic scaffold. The Alu- and 7SL RN A analogues induced morphological changes in approximately 20–30% of the cells by the 72^nd^ h of incubation. Moreover, incubation in the medium with total MCF-7 RN A or with a L1-RN A moiety analogue or with Lipofectamine without RN A caused condensation and detachment from the scaffold of less than 5% of the MCF- 7 cells.

The cells were incubated with the Alu- and 7SL RN A analogues, and their viability was analyzed using the MTT test to determine whether the morphological changes observed under the action of these analogues were caused by antiproliferative and proapoptotic processes.

**Table 1:** The effect of Alu-RNA and 7SL RNA analogues on the viability, asymmetry, cell membrane permeability, and mitochondrial transmembrane potential of MCF-7 cells

RN A^*^	Decrease in viability (MTT - index ± SD, %)^**^	Proapoptotic changes in membrane^***^			Mitochondrial transmembrane potential δΨ^****^, % of cells	
		Ann V-/PI-	Ann V+/PI-	Ann V+/PI+	without dissipation	with dissipation
		Viable cells, %	Apoptotic bodies, %	Secondary necrotic cells, %		
7SL RN A	19.0 ± 4.8	69.2	19.3	11.5	83.4	16.6
Alu-RN A	15.3 ± 6.5	68.7	13.8	17.5	85.6	14.4
RN A MCF-7	–2.8 ± 8.2	85.2	7.4	7.3	97.9	2.1

Lipofectamine	0 ± 2.5	89.9	6.8	3.3	99.7	0.3

^*^ Cells were transfected with 1μg/ml RNA in a complex with Lipofectamine.

^**^ Viability of cells incubated in the medium with Lipofectamine without RNA was taken as 100%.

^***^ Changes in the cell membrane were analyzed by flow cytofluorometry using annexin V (AnnV) conjugated to FITC and propidium iodide (PI).

^****^ Dissipation of the mitochondrial transmembrane potential was evaluated using flow cytofluorometry of cells stained with the mitochondrial dye JC-1 [23].

The data presented in Table 1 demonstrate that the Alu-RN A and 7SL RN A analogues cause a statistically significant reduction in MCF-7 cell viability upon transfection with Lipofectamine (*p * < 0.05). The observed morphological changes in conjunction with the reduction in viability under the action of the Alu- and 7SL RN A analogues indicate that transfection with these RN As leads to proapoptotic changes in cells.

We analyzed the changes in the mitochondrial transmembrane potential (δΨ) using the JC-1 indicator to evaluate, by an independent method, induction of the proapoptotic processes in MCF-7cells under the influence of Alu- and 7SL RN As. The indicator JC-1 forms aggregates in the mitochondria of viable cells, with the fluorescence spectrum shifted to the longer wavelengths (λ_max_ = 590 nm). Dissipation of the mitochondrial transmembrane potential δΨ is accompanied by a shift in the fluorescence spectrum maximum of the indicator to the green region (λ_max_ = 527 nm). The analysis of cell preparations by flow cytofluorometry in the presence of the JC-1 indicator made it possible to estimate the relative contribution of a cell population to proapoptotic changes the mitochondrial membrane [23, 25].

It was found that the reduction in MCF-7 cell viability under the action of the 7SL RN A analogue is accompanied by a reduction in the transmembrane potential δΨ in approximately 17% of the cells (Table 1). However, the action of 7SL RN A was not different from that of the Alu-RN A analogue (*p *> 0.05). Therefore, the data on the changes in the mitochondrial potential δΨ are consistent with the results of the viability analysis obtained using the MTT test, and with the evaluation of the depth of the morphological changes in the cells.

Transfection of cells with the Alu- and 7SL RN A analogues leads to the formation of cell-like structures exposing phosphatidylserine on the outer surface, as well as structures whose membrane is permeable to propidium iodide (apoptotic and secondary necrotic bodies). The overall contribution of the apoptotic and secondary necrotic bodies to the total population of cells transfected with the Alu-RN A analogue or 7SL RN A analogue was about 31% (Table 1).

It is known that the emergence of phosphatidylserine on the outer surface of the cytoplasmic membrane, detected by staining with annexin V, is one of the earliest biochemical signs of apoptosis [26]. Meanwhile, a decrease in the activity of mitochondrial and cytoplasmic oxidoreductases and a change in the NADH/ NADPH level, detectable using the MTT test [27], are characteristic of the late stages of apoptosis. Therefore, the differences in the cytotoxic action of Alu- and 7SL RN As, estimated from the reduction of the MTT -index (~ 15–19%) and from the induction of apoptotic processes by phosphatidylserine exposure and by plasma membrane permeability (~ 31%), can be attributed to the greater sensitivity of the approach using the annexin V/PI system.

Over all, these results suggest that analogues of both Alu-RN A and 7SL RN A reduce viability and induce proapoptotic changes in a MCF-7 cell subpopulation and that the effect of the Alu-RN A analogues is not significantly different from that of 7SL RN A at the level of the changes in the activity of cytoplasmic and mitochondrial dehydrogenases (MTT test), dissipation of the mitochondrial transmembrane potential δΨ, and by assessment of the depth of the morphological changes.

**Effect of Alu-RNA and RNA 7SL along with cytostatics on MCF-7 cell viability**

**Table 2:** Effect of Alu-RNA and 7SL RNA analogues on MCF-7 cell viability in the presence of cytostatic agents

Effector (IC_40_^*^)	Alu(+)-RN A		7SL(+)-RN A	
	MTT -index ± SD, %^**^	p^***^	MTT -index ± SD, %^**^	p^***^
Cisplatin (9.5 μM)	25.7 ± 7.7	0.004	20.0 ± 3.5	0.001
Cycloheximide (0.56 μM)	17.9 ± 6.7	0.010	14.9 ± 7.5	0.026
Interferon α (400 U/ml)	17.8 ± 7.6	0.022	26.5 ± 7.9	0.009
Methotrexate (33.3 μM)	11.5 ± 10.2	0.171	26.5 ± 8.4	0.011
Monensin (2.5 pM)	3.8 ± 6.3	0.352	10.8 ± 5.1	0.021
Tamoxifen (450 μM)	–1.2 ± 12.7	0.897	–12.1 ± 12.6	0.244
Actinomycin D (5.6 nM)	21.5 ± 21.2	0.232	-57.7 ± 22.6	0.031

^*^ The empirically obtained effector concentrations are indicated at which cell viability decreased by 40% after incubation (with Lipofectamine) for 72 hrs.

^**^ Additional decrease in the MTT-index in cells by the 72^nd^ h after transfection with RNA. Viability of cells incubated in the medium with Lipofectamine, with an effector at the indicated concentration, and without RNA was taken as 0%.

^***^ p value for the Student’s t-test.

Key processes, the inhibition or activation of which occurs upon transfection of cells with the Alu-RN A and 7SL RN A analogues, were characterized by a change in the MCF-7 viability upon the combined action of the analogues and a series of cytostatic agents (Table 2). The combined action of RN As and cellular process inhibitors was analyzed using such a cytostatic concentration in a culture medium at which MCF-7 cell viability was reduced by 40% (IC_40_) by the 72^nd^ h of incubation.

It is seen from the data in Table 2 that transfection of cells with the Alu- and 7SL RN A analogues enhances the cytotoxic action: for cisplatin by ~ 25 and 20%; for cycloheximide by ~ 18 and 15%; for interferon α by ~ 18 and 27%, respectively (*p * < 0.05). Therefore, transfection with the Alu- and 7SL RN A analogues caused an unidirectional and comparable magnitude effect on MCF-7 cells for this set of effectors.

The formation of unrepairable DNA crosslinks and suppression of replication and mitosis underlay the cytotoxic effect of cisplatin [28]. The additivity of cisplatin and Alu-RN A or 7SL RN A (Table 2) clearly indicates that the cytotoxic effects of this cytostatic agent and Alu-RN A or RN A 7SL are independent processes and the effects of these RN As are related directly neither to DNA replication nor to the activation of repair processes in MCF-7 cells.

The action of interferon α is based on the receptormediated transcriptional activation of interferon-induced genes, including the protein kinase PKR gene. PKR, in turn, is activated upon interaction with double- stranded RN A or with RN A comprising elongated hairpins, and it inhibits protein synthesis in the cell by phosphorylation of the translation initiation factor eIF2 [29]. Therefore, the additive action of the Alu- or 7SL RN A analogues and interferon α can be attributed to the fact that these RN As, having a developed secondary structure (*Fig. 1*), induce the PKR-dependent suppression of translation in cells treated with interferon α: on the other hand, PKR activation by double-stranded RN A serves as a signal for the induction of innate immune cell response cascades and, as a consequence, as an interferonogenic stimulus [29]. Therefore, the PKRdependent mechanism of the Alu- and 7SL RN A action provides manifold enhancement of the action of interferon α. At the same time, both Alu- and 7SL RN A cause the additive reduction of the MTT -index in interferon- stimulated cells, which is comparable to the reduction of viability upon combination of Alu- or 7SL RN A with cycloheximide or to the action of the RN As without interferon α (Table 1, Table 2). Moreover, a number of studies have shown that the action of Alu-RN A on different processes in mammalian cells is directly connected neither to the developed secondary structure of these RN As nor to the PKR activation [15, 21, 22]. Therefore, the PKR-dependent mechanism of action of structured RN As and the interferonogenic activity of such RN As only partially explain the induction of proapoptotic Alu- and 7SL RN A processes in MCF-7 cells.

7SL RN A, along with methotrexate and monensin, caused a significant decrease in the MTT -index (*p * < 0.05), but the variation of cell viability upon transfection with Alu-RN A, along with these cytostatic agents, was not statistically significant (Table 2). However, the decrease in the MTT -index of 7SL RN A in the presence of methotrexate or monensin was different from that induced by Alu-RN A along with these cytostatics (*p* < 0.05). These data demonstrate that the dihydrofolate reductase inhibitor methotrexate and ionophore monensin partially inhibit the cytotoxic effect of Alu- RN A, but not that of 7SL RN A.

An additional statistically significant reduction of viability (p > 0.05) in preparations of cells incubated in the medium with tamoxifen was not observed upon transfection with Alu-RN A or 7SL RN A (Table 2). Therefore, a conclusion can be drawn that tamoxifen partially suppresses the cytotoxic effect of both Alu- RN A and 7SL RN A on MCF-7 cells.

It is known that tamoxifen inhibits estrogen receptors, and that its effect on MCF- 7 cells is due to a change in the transcription of estrogen-dependent genes. Tamoxifen is also an effective modulator of interferon action. The combined effect of interferon and tamoxifen synergistically reduces MCF-7 cell viability and induces their massive death both in culture and in a xenograft model [30, 31]. Thus, the partial inhibition of the cytotoxic effect of the Alu- and 7SL RN A analogues on MCF-7 cells by tamoxifen confirms the assumption that the influence of these RN As on cell viability is not related to the potential interferonogenic properties of these structured RN As.

Actinomycin D, a DNA intercalator and an inhibitor of transcription and replication, completely inhibited the cytotoxic effect of the 7SL RN A analogue, while Alu-RN A, along with this cytostatic, caused no additional significant reduction of viability (Table 2). Taking into account that inhibition of replication with cisplatin did not reduce the effect of Alu- and 7SL RN A, it is possible to conclude that partial (in the case of Alu- RN A) and total (in the case of 7SL RN A) cessation of their cytotoxic action by actinomycin D is caused by the influence of these RN As on transcription in human cells. The data on the compensation of the Alu- and 7SL RN A cytotoxic effect by the transcription modulator tamoxifen (Table 2) support the conclusion that the modulation of nuclear DNA transcription is the key element of the action mechanism of both Alu-RN A and its closest homologue, 7SL RN A, on MCF-7 cell viability.

**Analysis of the variation in gene expression in MCF-7 cells under the influence of Alu- and 7SL RNA analogues**

Genes whose expression varies under the action of the Alu- and 7SL RN A analogues were determined by the whole transcriptome analysis of MCF-7 cell RN A using Illumina HT-12 microarrays. Cells incubated in the medium with Lipofectamine without RN A were used as a control.

It was found that transfection of MCF-7 cells with Alu-RN A results in an increase in the expression of 68 transcripts by 3 and more times and in a decrease in the expression of 87 transcripts. Transfection of cells with 7SL RN A increased the level of 45 genes by 3 and more times and lowered the level of 74 genes. Thirteen transcripts common to Alu- and 7SL RN A were revealed in groups of transcripts with increased expression. Twenty- five transcripts common to Alu- and 7SL RN A were detected in the groups with lowered expression. These data demonstrate that Alu-RN A and 7SL RN A cause variations in differing sets of transcripts, and they suggest that there are also differences in the specificity of the influence and, possibly, in the induction mechanisms of the pro-apoptotic processes in human cells. However, a detailed analysis of the variation in the expression of the pro- and anti-apoptotic factors allowed us to determine a number of key processes common to cells transfected with both Alu-RN A and 7SL RN A.

It is seen from the data presented in Tables 3 and* 4 *that products of interferon-inducible genes such as* OAS*, *ISG*, *IFIT, *or *STAT1 *are almost absent from the list of transcripts whose expression is increased to the greatest extent [32]. Moreover, the analysis of GOannotations in a group of 68 transcripts induced with Alu-RN A and in a group of 45 transcripts induced with 7SL RN A revealed no statistically significant (p < 10-4) increase in the contribution of groups of the interferon response genes and innate immune response genes (data not shown). These results, again, confirm the conclusion that induction of proapoptotic processes in human cells with the Alu-RN A and 7SL RN A analogues cannot be explained by the activation of PKR, the interaction with TLR-receptors, or by another mechanism associated with the interferonogenic action of these RN As.

Among the genes whose expression is increased under the action of both Alu-RN A and 7SL RN A (Tables 3, 4), *NUPR1 *stands out. It is known that the expression of the transcriptional regulator gene *NUPR1 *(encodes protein p8) is enhanced in response to various stress factors and results in cell resistance to chemotherapeutic agents, while a decrease in *NUPR1 *expression is accompanied by a suppression of cancer cell growth* in vitro *and *in vivo* [33, 34]. However, an increase in the level of *NUPR1 *mRN A also accompanies apoptotic changes in cancer cells [35].

The *DDIT3 *gene product, a CHOP transcription factor, is a key mediator of cell death in response to endoplasmic reticulum stress. Increased expression of this gene or microinjections of the CHOP protein cause dissipation of the mitochondrial transmembrane potential (δΨ), generation of reactive oxygen species, and apoptotic cell death (a detailed consideration is provided in the review [36]). Therefore, the observed increase in the expression of the *DDIT3 *gene in MCF-7 cells under the action of the Alu- and 7SL RN A analogues (Tables 3, 4) is an essential proapoptotic stimulus. The increase in the expression of *DDIT3 *(CHOP) and induction of apoptosis in response to endoplasmic reticulum stress can be directly induced by *NUPR1 *(p8) gene activation, as has been shown in the case of the cannabinoid-induced apoptosis of astrocytoma cells U87MG [35].

It should be mentioned that an increase in the level of* DDIT3 *mRN A, as well as *PSPH *and *MTHFD2 *mRN As, in MCF-7 cells under the influence of Alu-RN A or 7SL RN A (Tables 3, 4) was confirmed by random inspection of the results of a whole transcriptome analysis performed with the independent RT -PCR method (data not shown).

**Table 3:** MCF-7 cell transcripts whose level varies under the action of the Alu-RNA analogue

Transcript^*^	Identifier	Relative change in expression^**^	Annotation
Increase in expression			
**NUPR1**	NM_001042483	5.3	Nuclear protein, transcriptional regulator
PER3	NM_016831	5.1	Period homolog 3 (Drosophila)
**TXNIP**	NM_006472	4.7	Thioredoxin interacting protein
**ASNS**	NM_133436	4.5	Asparagine synthetase, transcript variant 1
ZNF773	NM_198542	4.3	Zinc finger protein 773
FAM119A	NM_001127395	4.1	Family with sequence similarity 119, member
ZNF750	NM_024702	4.1	Zinc finger protein 750
**PRRT2**	NM_145239	4.0	Proline-rich transmembrane protein 2
KCNE4	NM_080671	3.9	Potassium voltage-gated channel
C6ORF48	NM_001040437	3.9	Chromosome 6 open reading frame 48
AUH	NM_001698	3.8	AU RN A binding protein
**DDIT3**	NM_004083	3.8	DNA-damage-inducible transcript 3
**KRT81**	NM_002281	3.7	Keratin 81
**RNASE4**	NM_194430	3.6	Ribonuclease, RN ase A family 4
FBXO15	NM_152676	3.6	F-box protein 15
FLJ45244^***^	NM_207443	3.6	DICER1 antisense RN A 1 non-coding RN A
MTHFD2	NM_001040409	3.5	Methylenetetrahydrofolate dehydrogenase
Decrease in expression			
**FOXRED2**	NM_024955	0.15	FAD-dependent oxidoreductase domain containing 2
PPRC1	NM_015062	0.19	Peroxisome proliferator-activated receptor gamma, coactivator-related 1
**CHP**	NM_007236	0.21	Calcium binding protein P22
**PHLDA2**	NM_003311	0.21	Pleckstrin homology-like domain, family A, member 2
TMEM158	NM_015444	0.21	Transmembrane protein 158
ATN1	NM_001007026	0.22	Atrophin 1 (ATN 1)
**DLK2**	NM_206539	0.23	Delta-like 2 homolog (Drosophila)
HPS1	NM_182639	0.23	Hermansky-Pudlak syndrome 1
TMEM214	NM_017727	0.23	Transmembrane protein 214
MED24	NM_014815	0.24	Mediator complex subunit 24
**PLEC1**	NM_000445	0.24	Plectin 1, intermediate filament binding protein 500 kDa
ZYX	NM_003461	0.24	Zyxin
ACD	NM_022914	0.25	Adrenocortical dysplasia homolog (mouse)
PCDH7	NM_002589	0.25	Protocadherin 7 (PCDH7)
RDH10	NM_172037	0.25	Retinol dehydrogenase 10 (all-trans)
**GPX2**	NM_002083	0.26	Glutathione peroxidase 2 (gastrointestinal)

^*^ Transcripts annotated in the RefSeq database (accessions NM, NR). The transcripts whose expression changed under the action of both Alu-RNA and 7SL RNA are shown in **bold**.

^**^ Variation in the transcript amount in cells treated with Alu-RNA relative to the control cells treated with Lipofectamine.

^***^ The Illumina HT-12 probe sequence for the FLJ45244 gene coincides with the DICER-AS1 sequence (NR_015415).

**Table 4:** MCF-7 cell transcripts whose level varies under the action of the 7SL RNA analogue

Transcript^*^	Identifier	Relative change in expression^**^	Annotation
Increase in expression			
**NUPR1**	NM_001042483	4.5	Nuclear protein, transcriptional regulator
**TXNIP**	NM_006472	4.3	Thioredoxin interacting protein
**PRRT2**	NM_145239	4.3	Proline-rich transmembrane protein 2
PSPH	NM_004577	4.2	Phosphoserine phosphatase
**ASNS**	NM_133436	3.8	Asparagine synthetase, transcript variant 1
KY	NM_178554	3.8	Kyphoscoliosis peptidase
FABP6	NM_001445	3.7	Fatty acid binding protein 6, ileal
**DDIT3**	NM_004083	3.6	DNA-damage-inducible transcript 3
CTSK	NM_000396	3.6	Cathepsin K
**KRT81**	NM_002281	3.6	Keratin 81
PFAAP5	NM_014887	3.4	Phosphonoformate immuno-associated protein 5
NT5E	NM_002526	3.4	5'-Nucleotidase, ecto (CD73)
ARL3	NM_004311	3.4	ADP-ribosylation factor-like 3
**ULBP1**	NM_025218	3.4	UL16 binding protein 1
BACE2	NM_138992	3.4	Beta-site APP-cleaving enzyme 2
**RNASE4**	NM_194431	3.3	Ribonuclease, RN ase A family 4
Decrease in expression			
**FOXRED2**	NM_024955	0.16	FAD-dependent oxidoreductase domain containing 2
**GPX2**	NM_002083	0.16	Glutathione peroxidase 2 (gastrointestinal)
**TUBB2A**	NM_001069	0.17	Tubulin, beta 2A
**PLEC1**	NM_000445	0.19	Plectin 1, intermediate filament binding protein
ZC3HAV1	NM_024625	0.20	Zinc finger CCC H-type, antiviral 1
SLC35C1	NM_018389	0.21	Solute carrier family 35, member C1
**NCOR2**	NM_001077261	0.21	Nuclear receptor co-repressor 2
PIGW	NM_178517	0.22	Phosphatidylinositol glycan anchor biosynthesis, class W
**MUC1**	NM_001044391	0.22	Mucin 1, cell surface associated
**OPA3**	NM_025136	0.23	Optic atrophy 3 (autosomal recessive, with chorea and spastic paraplegia)
PDPK1	NM_002613	0.23	3-Phosphoinositide dependent protein kinase-1
SLC29A3	NM_018344	0.23	Solute carrier family 29 (nucleoside transporters), member 3
HCFC1	NM_005334	0.24	Host cell factor C1 (VP16-accessory protein)
FAHD1	NM_001018104	0.24	Fumarylacetoacetate hydrolase domain containing 1 (FAHD1)
PARP12	NM_022750	0.24	poly (ADP-ribose) polymerase family, member 12
LRRC14	NM_014665	0.25	Leucine rich repeat containing 14

^*^ Transcripts annotated in the RefSeq database (accessions NM, NR). The transcripts whose expression changed under the action of both Alu-RNA and 7SL RNA are shown in **bold**.

^**^ Variation in the transcript amount in cells treated with Alu-RNA relative to the control cells treated with Lipofectamine.

Expression of the *FOXRED2 *gene is reduced under the action of both Alu- and 7SL RN A (Tables 3, 4). The* FOXRED2 *gene product flavoprotein ER FAD participates in the transport of proteins from the endoplasmic reticulum into the cytoplasm. A reduction in the expression of this gene is associated with activation of proteotoxic stress in the endoplasmic reticulum [37]. Another sign of activation of the endoplasmic reticulum stress response is an increase in asparagine synthetase* ASNS *gene expression, whose transcription is activated by the CC AAT/enhancer binding protein CHOP [38].

Therefore, the decrease in *FOXRED2 *expression, observed simultaneously with an increase in the level of* NUPR1 *(p8), *DDIT3 *(CHOP), and *ASNS*, suggests that induction of the proapoptotic processes in MCF-7 cells under the influence of Alu- and 7SL RN As is associated with modulation of the transcription of the key cellular factors of the endoplasmic reticulum stress response.

A new mechanism for the development of geographic atrophy of the retina has recently been proposed, which is based on a decrease in *DICER1 *expression in the epithelial cells and enhanced expression of Alu- RN A [21]. Subretinal transfection of cells with a construct encoding 7SL RN A, as well as with a 7SL RN A analogue, did not lead to degeneration of the retinal pigment epithelium in mice in contrast to transfection with Alu-RN A [21, 22]. It has been suggested that the cytotoxic effect of Alu-RN A on retinal pigment epithelial cells is associated with unidentified properties of Alu-RN A, and that the mechanism of action is associated with the generation of reactive oxygen species by mitochondria [22].

**Fig. 2:**
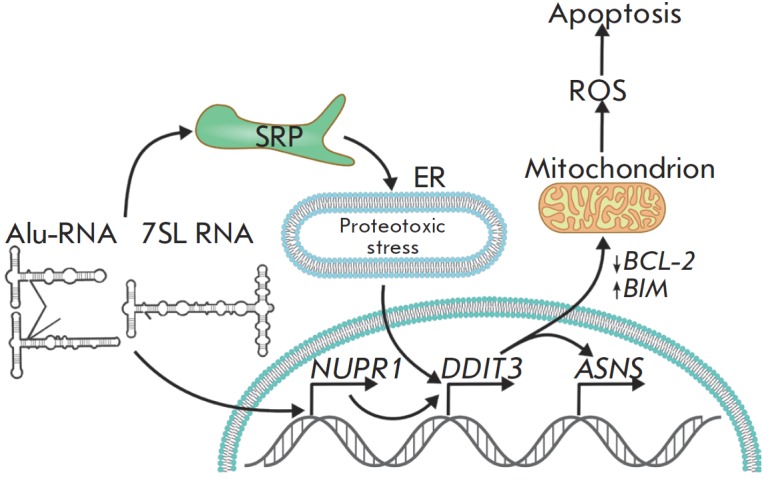
A scheme of the supposed mechanism of induction of proapoptotic processes in MCF-7 cells transfected with Alu- and 7SL RNA analogues. Transfection of cells with Aluand 7SL RNA analogues is accompanied by an increase in the expression of the *NUPR1 *(p8) transcription regulator gene which activates the transcription of *DDIT3 *(CHOP) [35]. The increase in the *DDIT3 *transcription factor expression causes apoptotic changes in the mitochondrial outer membrane through a mechanism including a reduction in the *BCL-2 *transcription and activation of *BIM *transcription. CHOP (*DDIT3*)-induced apoptosis is accompanied by the generation of reactive oxygen species (ROS) [36]. The increase in *DDIT3 *expression can occur as a response to the endoplasmic reticulum stress caused by the interaction of Alu- and 7SL RNAs with SRP proteins – failure in the protein transport through the ER membrane. Endoplasmic reticulum stress is accompanied by an increase in the expression of the asparagine synthetase (*ASNS*) gene, whose transcription is activated by CHOP [38]

Our data demonstrate that both Alu- and 7SL RN As cause comparable changes in the mitochondrial transmembrane potential of MCF-7 cells (Table 1). Consequently, both Alu-RN A and 7SL RN As induce similar changes in the mitochondrial membrane at least in MCF-7 cells. The analysis of the action of Alu- and 7SL RN As, along with actinomycin D and tamoxifen, on MCF-7 cell viability revealed that the cytotoxic effect of these RN As was caused by transcription modulation. The data on the variation of gene expression (Tables 3, 4) demonstrate that transfection of cells with Alu-RN A or 7SL RN A analogues is accompanied not only by a nonspecific response to exogenous RN A, an increase in the levels of *RNASE4 *ribonuclease mRN A and *NT5E *5’-ectonucleotidase mRN A, but also by the emergence of proapoptotic stimuli: *NUPR1*, *DDIT3*,* FOXRED2*. While *NUPR1 *gene expression is induced in response to a wide range of stress factors, *DDIT3* and *FOXRED2 *are specifically related to the endoplasmic reticulum stress response. The *DDIT3 *gene product, the CHOP protein, is the key apoptosis inducer in the proteotoxic ER stress response. The obtained data suggest a mechanism of Alu- and 7SL RN A proapoptotic action which includes activation of the transcription of the *NUPR1 *(p8) and proapoptotic *DDIT3 *genes. The product of the latter, CHOP, induces apoptosis through the mitochondrial pathway in a MCF-7 cell subpopulation (*Fig. 2*).

Since 7SL RN A is a component of the signal recognition particle and Alu-RN A is capable of interacting with the proteins SRP9/14, it can be assumed that activation of the endoplasmic reticulum stress response with Alu- and 7SL RN A analogues is caused by a malfunction of this very component translational machinery of human cells.

## CONCLUSIONS

It was found previously that an increase in the expression of Alu-RN A in human cells causes the suppression of DNA replication, inhibits translation, and exerts an antiproliferative effect. Our data indicate that nuclear DNA transcription is the key process that mediates the decrease in the viability of MCF-7 human adenocarcinoma cells under the action of both Alu-RN A and 7SL RN A analogues. However, no activation of the expression of interferon-inducible genes is observed. Meanwhile, transfection of MCF-7 cells with Alu-RN A or 7SL RN A is accompanied by changes in the expression of a number of genes, including *NUPR1*, *DDIT3*,* FOXRED2, *and *ASNS*. Variation in the transcription of these genes is known to be associated with the complex cell response to ER stress, which is capable of inducing the formation of reactive oxygen species and cell death through the mitochondrial apoptosis pathway. Activation of the ER stress response under the influence of Alu- and 7SL RN A analogues is presumably associated with SRP malfunction in cells.

On the whole, our results and published data indicate that Alu-RN A is not only a marker, but also a mediator of cell stress signals.

## Abbreviations

FITC: fluorescein-5-isothiocyanate
ER: endoplasmic reticulum
MTT: 3-(4,5-dimethyl-2-thiazolyl)-2,5-diphenyl-2H-tetrazolium bromide
JC: 1 – 5,5’,6,6’-tetrachloro-1,1’,3,3’-tetraethylbenzilimidazolocarbocyanineiodide
SRP: signal recognition particle
